# High INtensity Interval Training In pATiEnts with intermittent claudication (INITIATE): protocol for a multicentre, proof-of-concept, prospective interventional study

**DOI:** 10.1136/bmjopen-2020-038825

**Published:** 2020-07-06

**Authors:** Sean Pymer, Amy Harwood, Said Ibeggazene, Gordon McGregor, Chao Huang, Maureen Twiddy, Adam R Nicholls, Lee Ingle, Sean Carroll, Judith Long, Marjorie Rooms, I C Chetter, On behalf of INITIATE investigator group

**Affiliations:** 1 Academic Vascular Surgical Unit, Hull York Medical School, Hull, UK; 2 Centre for Sport, Exercise and Life Sciences, Coventry University, Coventry, New South Wales, UK; 3 Department of Sport, Health and Exercise Science, University of Hull, Hull, UK; 4 Department of Cardiac Rehabilitation, Centre for Exercise and Health, University Hospitals Coventry and Warwickshire NHS Trust, Coventry, UK; 5 Warwick Clinical Trials Unit, Warwick Medical School, University of Warwick, Coventry, UK; 6 Institute of Clinical and Applied Health Research, Hull York Medical School, University of Hull, Hull, UK; 7 Hull, UK

**Keywords:** vascular surgery, rehabilitation medicine, vascular medicine

## Abstract

**Introduction:**

The first-line recommended treatment for patients with intermittent claudication (IC) is a supervised exercise programme (SEP), which includes a minimum of 2-hours of exercise per week over a 12-week period. However, provision, uptake and adherence rates for these SEP programmes are poor, with time constraints cited as a common participant barrier. High-intensity interval training (HIIT) is more time-efficient and therefore has the potential to overcome this barrier. However, evidence is lacking for the role of HIIT in those with IC. This proof-of-concept study aims to consider the safety, feasibility, tolerability and acceptability of a HIIT programme for patients with IC.

**Methods and analysis:**

This multicentre, single-group, prospective, interventional feasibility study will recruit 40 patients with IC, who will complete 6 weeks of HIIT, 3 times a week. HIIT will involve a supervised programme of 10×1 min high-intensity cycling intervals at 85%–90% peak power output (PPO), interspaced with 10×1 min low intensity intervals at 20%–25% PPO. PPO will be determined from a baseline cardiopulmonary exercise test (CPET) and it is intended that patients will achieve ≥85% of maximum heart rate from CPET, by the end of the second HIIT interval. Primary outcome measures are safety (occurrence of adverse events directly related to the study), programme feasibility (including participant eligibility, recruitment and completion rates) and HIIT tolerability (ability to achieve and maintain the required intensity). Secondary outcomes include patient acceptability, walking distance, CPET cardiorespiratory fitness measures and quality of life outcomes.

**Ethics and dissemination:**

Ethical approval was obtained via a local National Health Service research ethics committee (Bradford Leeds – 18/YH/0112) and recruitment began in August 2019 and will be completed in October 2020. Results will be published in peer-reviewed journals and presented at international conferences and are expected to inform a future pilot randomised controlled trial of HIIT versus usual-care SEPs.

**Trial registration number:**

NCT04042311; Pre-results.

**Strengths and limitations of this study**

- This study will assess the safety and feasibility of a novel, pragmatic high-intensity interval training programme for patients with intermittent claudication.
- It will also consider acceptability of the programme via qualitative methods of patient feedback.
- As a limitation, due to the single-group design it is not possible to identify if patients who choose to take part in this study are simply those who would have also chosen to take part in a usual-care exercise programme.

## Introduction

Peripheral arterial disease (PAD) is caused by atherosclerotic lesions in the arteries supplying the lower limbs, reducing blood flow.^1^ PAD is relatively common, age-dependent and increasing in its prevalence. In 2010, it was estimated that PAD affected 202 million people globally, with those aged 75 or older having an approximately eightfold risk compared with those aged less than 60.^2 3^ Compounded by population ageing and an increase in the prevalence of diabetes, it was estimated that the number of people living with PAD increased over the previous decade by 13% and 29% in high-income and low-middle-income countries, respectively.^3^

Symptomatic PAD typically presents as intermittent claudication (IC), defined as a reproducible ambulatory leg pain, in the calf and/or thigh and/or buttocks, caused by an oxygen supply–demand imbalance, relieved by rest.^4 5^ As such, IC negatively impacts on walking ability, functional capacity, quality of life (QoL) and daily activities, while also leading to a markedly increased mortality risk.^6–11^ The recommended treatment strategy for IC is non-invasive and includes pharmacological risk factor management and exercise therapy, via a supervised exercise programme (SEP).^12–14^ SEPs should consist of a minimum of 2-hours of exercise per week for a 12-week period, with patients encouraged to exercise to the point of maximal pain.^12^ SEPs are supported by high-quality evidence for their clinical and cost-effectiveness,^15^ with evidence also suggesting that SEPs are equal to primary stenting for symptomatic improvement, which is maintained for a year after programme completion.^16 17^

Despite the irrefutable evidence for the benefit of SEPs, just 39% of UK vascular centres provide access to one,^18^ and for those that do, uptake and completion rates are suboptimal. One review demonstrated that only 25% of screened patients are recruited to a programme,^19^ with time cited as the most common barrier for participation.^20^ Furthermore, the current recommendations for SEPs appear to adopt a ‘one size fits all’ approach which is not based on any objective measure of functional capacity, potentially limiting physiological and symptomatic benefits. One alternative that is both time-efficient and prescribed based on the gold-standard measure of cardiopulmonary exercise testing (CPET), is high-intensity interval training (HIIT). HIIT, therefore, has the potential to overcome the previously cited programme-related drawbacks of traditional SEP. HIIT has demonstrated similar or superior benefits, when compared with traditionally prescribed exercise, in patients with coronary artery disease, chronic heart failure, hypertension, obesity and metabolic syndrome characteristics.^21–24^ HIIT has been highlighted as a potentially preferred treatment option in those with IC, though the evidence in this population is much more limited.^20^ Initial systematic review evidence has indicated that HIIT has the potential to provide clinical and symptomatic benefits, though there was significant heterogeneity between published studies in terms of HIIT modality, frequency, intensity and duration.^25^ The authors recommended that future appropriately designed studies consider shorter-term and low-volume HIIT programmes for patients with IC.

Therefore, the aim of this multicentre proof-of-concept study is to consider the safety, tolerability, feasibility and acceptability of a short-term, low-volume HIIT programme in those with IC.

## Methods and analysis

High Intensity Interval Training In pATiEnts With Intermittent Claudication (INITIATE) is a pragmatic, single-group, multicentre and prospective interventional proof-of-concept study. The study design and inclusion/exclusion criteria have been informed by a previous, single-centre study including 30 patients.^26^

For this study, participants will be recruited consecutively and perform 6 weeks of HIIT. Study interventions and outcome assessments will be conducted by research staff that due to the nature of the study cannot be blinded. This protocol adheres to the Standard Protocol Items: Recommendations for Clinical Trials (SPIRIT) guidelines, and we used the SPIRIT checklist when writing this protocol.^27 28^

### Setting

INITIATE will be conducted at two UK centres; 1. The Academic Vascular Surgical Unit, Hull Royal Infirmary, Kingston-Upon-Hull and 2. Atrium Health, Centre for Exercise and Health, Coventry and Warwickshire Hospitals National Health Service (NHS) Trust, Coventry. Sponsorship is provided by Hull University Teaching Hospitals NHS Trust and funding provided by the National Institute for Health Research, Research for Patient Benefit programme. Recruitment commenced in August 2019, with a recruitment target of 40 patients (20 per site). Recruitment is anticipated to be completed by October 2020.

### Study registration

The study was prospectively registered on ClinicalTrials.gov and the study registration data set is given in table 1. Any amendments required to this protocol will seek approvals from the research ethics committee and will be outlined (with reasons) in the final published report.

**Table 1:** Study registration items

Data category	Information
Primary registry and identifying number	ClinicalTrials.gov NCT04042311 (Workstream 2)
Date of registration in primary registry	01/08/2019
Source of monetary or material support	National Institute for Health Research, Research for Patient Benefit programme
Primary Sponsor	Hull University Teaching Hospitals NHS Trust
Contact for public queries	JL (Judith.Long@hey.nhs.uk)
Contact for scientific queries	SP (Sean.Pymer@hey.nhs.uk)
Public title	High INtensity Interval Training In pATiEnts with intermittent claudication (INITIATE)
Scientific title	INITIATE: a multicentre, proof-of-concept, prospective interventional study
Countries of recruitment	UK
Health condition or problem studied	Intermittent claudication
Intervention	High-intensity interval training
Key inclusion and exclusion criteria	Ages eligible for the study: ≥18 years Sexes eligible for the study: all Accepts healthy volunteers: no
	Inclusion criteria: Community dwelling adults aged 18 or over. ABPI <0.9 at rest or a drop of more than 20 mm Hg after exercise testing Ability to walk unaided English speaking and able to comply with exercise instructions
	Exclusion Criteria: Unable to provide informed consent Critical limb threatening ischaemia/rest pain/tissue loss Active cancer treatment Significant comorbidities precluding safe participation in exercise testing and / or training according to the American College of Sports Medicine guidelines^28^ Resting/uncontrolled tachycardia (>100 bpm) and/or resting/uncontrolled hypertension (systolic blood pressure >180 mm Hg or diastolic blood pressure >100 mm Hg) Symptomatic hypotension
Additional exclusion criteria:	Exercise-induced myocardial ischaemia or significant haemodynamic compromise (manifesting as anginal symptoms, significant ECG changes or an abnormal blood pressure response).
Study type	Interventional
	Allocation: single group assignment
	Primary purpose: Treatment
Date of first enrolment:	12/08/2019
Target sample size:	40 patients
Recruitment status:	Recruiting
Primary outcomes:	Safety: occurrence of adverse and serious adverse events Feasibility: eligibility, recruitment and completion rates Tolerability: assessing reasons for withdrawal, and identifying ability to reach and maintain the required intensity.
Secondary outcomes:	Acceptability: patient feedback via semistructured interview Efficacy: pain-free and maximal walking distance Quality of life Cardiorespiratory measures Ankle brachial pressure index

ABPI, Ankle-Brachial Pressure Index.

### How the sample will be selected

This study will recruit patients with IC secondary to PAD referred to a usual-care SEP, with a confirmed diagnosis of IC by resting and/or postexercise ankle-brachial pressure index (ABPI) and/or documented significant atherosclerosis on radiological imaging.

#### Inclusion criteria

- Aged≥18 years.
- ABPI<0.9 at rest or a systolic pressure drop of ≥20 mm Hg at the ankle after exercise testing.
- Ability to walk unaided.
- English speaking and able to comply with exercise instructions.

#### Exclusion criteria

- Unable to provide informed consent.
- Critical limb threatening ischaemia/rest pain/tissue loss.
- Active cancer treatment.
- Significant comorbidities precluding safe participation in exercise testing and/or training according to the American College of Sports Medicine (ACSM) guidelines.^29^
- Resting/uncontrolled tachycardia (>100 bpm) and/or resting/uncontrolled hypertension (systolic blood pressure>180 mm Hg or diastolic blood pressure>100 mm Hg).
- Symptomatic hypotension.

#### Additional exclusion criteria

Following baseline CPET, patients will be withdrawn and prevented from continuing their involvement in the study if there is any evidence of:

- Exercise-induced myocardial ischaemia or significant haemodynamic compromise (manifesting as anginal symptoms, significant ECG changes or an abnormal blood pressure response).

#### Study procedures

**Figure 1:**
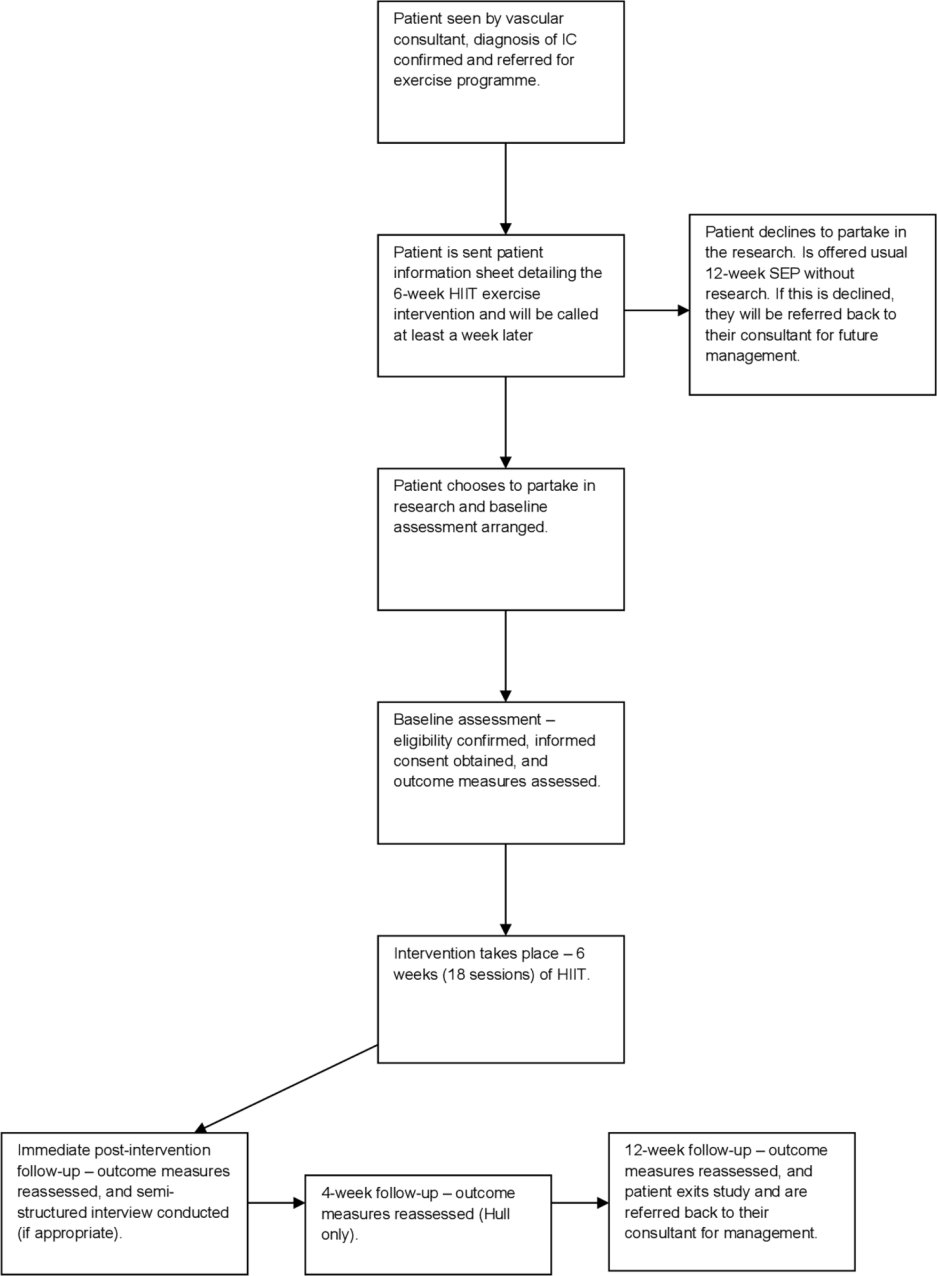
Participant study flow. HIIT, high-intensity interval training; IC, intermittent claudication; SEP, supervised exercise programme

The participant pathway for the study is shown in figure 1. Briefly, patients who are deemed eligible for a usual SEP will be referred to the research team and their medical history reviewed to determine potential eligibility for INITIATE. Those appearing to meet the eligibility criteria will be sent an invitation letter and patient information sheet. Patients will then be contacted at least a week later via telephone to give them the opportunity to ask any questions and confirm if they are willing to participate. Those who decide to participate will be asked to attend a baseline visit where eligibility will be confirmed before informed consent is obtained. Those who decline the study will be offered SEP as per usual care.

Acceptability of the study and intervention will be assessed using qualitative interviews. The participant information sheet and consent form will include a clause that outlines the conduction of an interview with a subset of patients. The interview is optional, and participants can decline to be interviewed. Baseline and follow-up procedures will include a full and detailed medical history, medication and symptom review, assessment of ABPI and a Gardner-Skinner graded treadmill test,^30^ followed by postexercise ABPI. For those who are confirmed eligible, spirometry and CPET will be subsequently undertaken. QoL measures will also be collected using the Medical Outcomes Study Short-Form 36 (SF-36) and the Kings College Hospitals Vascular QoL (VascuQoL) Questionnaires, both of which have demonstrated good reliability and validity in this patient population.^31 32^ Following baseline CPET, exercise ECG and haemodynamic response will be evaluated to reassess eligibility to undergo HIIT. Those who exhibit exercise-induced ischaemia or an abnormal haemodynamic response to volitional exhaustion will be withdrawn from the study and referred back to the vascular consultant/required specialty as appropriate. Given CPET is not part of routine care, all patients will sign informed consent prior to undergoing it. Measurements will be taken before starting the programme (baseline/week 0), immediately after completing the programme (week 6), then 12 weeks later (week 18). A further follow-up will be conducted 4 weeks (week 10) after programme completion at the Hull site only.

### Intervention

This study will adapt a pragmatic and flexible HIIT protocol, based on a similar protocol currently being investigated in those with coronary artery disease.^33^ Patients will attend three HIIT sessions per week for a period of 6 weeks, totalling 18 sessions. If participants miss sessions, the intervention period can be extended for up to two additional weeks to allow these sessions to be completed. Those not completing 18 sessions over the extended 8-week period will be deemed to have satisfactorily completed the intervention as long as they have undertaken >80% of the HIIT sessions (ie, ≥15 out of 18 sessions). All patients completing the allotted 6–8 weeks for the intervention (regardless of whether they have completed ≥or <15 sessions) will be followed up. Those selecting to discontinue the intervention prematurely will be withdrawn, but the information collected up to their withdrawal will be retained and may still be used.

The intervention will be performed using a cycle ergometer (Wattbike Trainer, Wattbike, Nottingham, UK), with exercise prescription based on the peak workload achieved during the cycle CPET at baseline. Variations from high to low intensity cycling will be achieved by altering the cycle cadence (rpm). Although walking is often the recommended mode of exercise for those with IC and a treadmill based HIIT programme has been previously considered,^34 35^ a cycle was chosen for the current investigation for a number of reasons. First, the use of a treadmill may preclude patients from reaching their prescribed HIIT training zones due to limiting claudication pain. Stationary cycling may also reduce the risk of falls, given the balance limitation often experienced by patients with IC.^36^ In addition, it has been demonstrated that the limiting symptoms during treadmill walking are often experienced in the leg, predominantly the calf, whereas the limiting symptoms during cycling are much more varied.^37^ Finally, it has also been noted that cycle testing is better tolerated than treadmill testing in those with IC, which is important considering that the HIIT training zone requires the patient to exercise intermittently to near-peak exertion levels.^38^

Our HIIT work to rest ratio will be 1:1 (1 min high-intensity work interspaced with 1 min of low-intensity work), with patients completing 10 intervals for an overall exercise session time of 20 min. If required, a titrated introduction to the HIIT programme will be used with fewer exercise intervals being completed in the first 2 weeks. Patients will also be allowed to complete less than 10 intervals for longer than the first 2 weeks if required but will be encouraged to complete 10 as soon as possible thereafter. HIIT workloads will be set at 85%–90% of the peak power output achieved during the baseline CPET. Application of this workload aims to achieve 85%–100% peak heart rate (HRpeak) from CPET by the end of the second interval. Our personal experience with cardiac patients has demonstrated that patients may exceed their HRpeak (from baseline CPET) during HIIT sessions. This is also likely to be the case for those with IC, especially those who are unable to achieve a maximal effort CPET. We will adopt a pragmatic approach to this by allowing it to occur without adjusting workload but will monitor on a case-by-case basis and reduce cycling intensity when it is deemed appropriate. We will also record these occurrences to allow appropriate reporting. All sessions will be preceded and followed by a 10 min warm-up and cool-down as is standard practice for exercise rehabilitation for older adults with chronic disease.

### Outcome measures

#### Primary outcomes

The primary outcomes for this study are safety, feasibility and tolerability.

**Safety** will be assessed by determining the occurrence of any adverse or serious adverse events related to the intervention or study procedures. These events will be recorded in accordance with the Good Clinical Practice (GCP) decision tree for adverse event reporting and where applicable events will be reported to the sponsor and/or research ethics committee.

**Feasibility** will be assessed by considering eligibility (*n* = eligible/screened), recruitment (*n* = recruited/eligible) and adherence (*n* = completed/recruited). As such, the number of patients screened, recruited, commencing and completing (either satisfactorily or unsatisfactorily) the HIIT programme will be monitored at each site.

**Tolerability** will be assessed by considering reasons for withdrawal (ie, if they are related to the intervention) and identifying the number of patients able to reach and maintain the required intensity (ie, ≥85% HRMax by the end of the second interval) for the full 10 intervals. Tolerability will also assess whether patients can complete the full 10 intervals by the end of the second week.

#### Secondary outcomes

Secondary outcome measures include, acceptability, pain-free and maximal walking distance, ABPI, QoL and cardiorespiratory measures, collected during CPET.

#### Acceptability

will be assessed by conducting semistructured interviews at both sites using a sample of patients in three groups:

Group 1: Patients who are eligible for the study but decide not to participate (non-consenters). The interviews will explore reasons why patients chose not to participate in the study and whether study material could be amended to be more appealing. As these patients have declined participation in the study, they will sign an interview specific consent form.

Group 2: Those who agree to participate in, and complete, the exercise programme (completers). The interviews will explore patient’s experiences of the HIIT programme, how acceptable they found it, whether they enjoyed it and whether they would be willing to undertake it again. They will also be asked to provide information related to potential barriers to participation in the programme and study, and any changes they may feel are required.

Group 3: Those who agree to participate but discontinued after at least one session (withdrawals). Patients will be asked about their reasons for discontinuation and what, if anything, could have been modified to prevent withdrawal from the study.

An interview topic guide with a predetermined set of open questions will be used but the interviews will be flexible to allow the interviewer to ask further probing questions based on patient responses, and for patients to raise issues not explicitly covered by the topic guide. All interviews will be audio recorded using a Dictaphone, transcribed verbatim and anonymised.

#### Pain-free and maximal walking distance

Pain-free and maximal walking distance will be determined using the Gardner/Skinner treadmill test which starts at 2.0 mp/hour and 0% gradient, with gradient increasing by 2% every 2 min, while the speed remains constant, up to a maximum of 15 min. For those unable to walk on the treadmill at 2.0 mp/hour the speed will be reduced, but this speed will remain consistent at all follow-up visits to ensure standardisation. Patients will indicate when they begin to feel IC pain, which will be recorded as pain-free walking distance and the patient will continue until the pain is too severe and they need to stop, which will be recorded as maximal walking distance. Patients able to walk for 15 min will be excluded.

#### Quality of life

QoL will be assessed with both a generic and disease specific questionnaire. The SF-36 will be used as it is recommended as the most appropriate generic tool for those with lower limb ischaemia.^39^ The SF-36 gives a scoring profile across eight domains, ranging from 0 to 100, with 0 indicating worst possible health and 100 best possible health. Scales can also be combined to create a physical and mental component summary.

The disease-specific questionnaire will be the VascuQoL which was designed for use in studies involving patients with lower limb ischaemia. It contains 25 items subdivided into 5 domains, which are rated on a 7-point scale with 1 representing the worst score and 7 the best. A sum score is also calculated by dividing the total score by 25.

#### Cardiorespiratory measures

Cardiorespiratory function will be assessed at each time point using an individualised ramp based cycle CPET, conducted in accordance with international guidelines.^40 41^ Patients will be screened for contraindications to CPET and continuously monitored for indications for termination as per the ACSM guidelines.^29^ The CPET will be preceded by a 3 min period of rest on the bike to obtain resting measurements followed by a 3 min reference period of unloaded cycling followed by a progressive individualised ramp protocol designed to elicit volitional exhaustion within 8–12 min, concluding with a recovery period.^41^ Patients will be encouraged to maintain 65–70 rpm throughout the test until they are limited by volitional fatigue. Monitoring will be via 12-lead ECG, blood pressure, oxygen saturation and rating of perceived exertion (RPE). Attainment of a maximal effort will be considered if the patient achieves 2 out of the following three criteria; achieving≥85% age-predicted maximum heart rate, a respiratory exchange ratio>1.10 and an RPE >17.^42^ However, based on a previous study, ∼25% of patients with IC are unable to achieve this, meaning it will not be applied as an exclusion and patients will continue in the study, regardless of whether it is achieved.^26^ Breath-by-Breath gas analysis will be conducted (MedGraphics Ultima2 Medgraphics, St Paul, Minnesota, USA or Ergostick, LoveMedical, Manchester, UK) to allow determination of a number of cardiorespiratory fitness parameters.

#### Ankle-Brachial Pressure Index

The systolic blood pressure will be measured bilaterally in the brachial, dorsalis pedis and posterial tibial arteries using a hand-held doppler and appropriately sized sphygmomanometer, with ABPI determined by dividing the higher ankle pressure of each leg with the highest arm pressure. Patients will be deemed eligible if they have an ABPI of <0.9 or a postexercise systolic blood pressure drop at the ankle of≥20 mm Hg.

### Sample size

As this feasibility proof-of-concept study does not aim to make any statistical comparison nor estimate an SD for future power calculations, there is no formal sample size requirement. We aim to recruit 20 patients from each site over the recruitment period, for a total of 40 patients.

### Data collection and management

Data will be collected by the study team across 3/4 time points dependant on site. Data will be collected continuously for the qualitative study, based on the time at which patients decline, withdraw from or complete the intervention, until the point of data saturation. Data will be collected and retained in accordance with the General Data Protection Regulation (2018). All patients will be given a study code to ensure anonymity. Data will be stored via paper case report forms (CRFs) in code-secured research offices at the vascular laboratory in Hull Royal Infirmary and Coventry and Warwickshire University Hospital respectively with the same identification code. These CRFs will be periodically scanned and sent to the team at Hull Royal Infirmary, who will manage the electronic and physical database, via email with end-to-end encryption. This database will be stored on a computer in the code-secured research office that is password protected and has both antivirus and firewall software. Only authorised members of the research team will have access to the patient data and transfer of data will be via Trust encrypted, password-protected remote storage devices or secure nhs.net mail. Only authorised members of the research team will have access to the final dataset which will be stored for 5 years following study completion.

### Data analysis

Where applicability allows, the study will be reported in accordance with the Consolidated Standards of Reporting Trials (2010) statement extension to pilot and feasibility studies.^43^ Descriptive statistics will be reported for our feasibility, tolerability and safety (proof-of-concept) outcomes. Descriptive statistics for our secondary outcomes will be reported to inform potential future studies in terms of clinical and QoL outcome measures.

The qualitative data will be analysed using an inductive thematic analysis, whereby themes are identified from within the data.^44^ The researcher will read and reread the transcripts to identify patterns of responses within the data that are related to the research question and can be grouped together under a theme heading. The approach will be inductive, which means that the themes are data driven, thus emerging from the data, and do not fit into a pre-existing coding frame.^44^

### Patient and public involvement

The background patient and public involvement (PPI) work for this study was supported by a grant from the NIHR Research Design Service Yorkshire and the Humber. Consequently, two focus group sessions, each involving five patients with a confirmed diagnosis of IC and experience of undertaking a standard SEP, were conducted which informed the design of this study. In addition, this PPI group is committed to continuous contribution during the research study, with the chair of the PPI group invited to attend all trial steering committee meetings. We also aim to hold 3–4 PPI meetings over the course of the study to aid with addressing potential recruitment or retention issues and aid with dissemination of the study findings.

## Ethics and dissemination

Protocol approval was obtained via a local NHS research ethics committee (Bradford Leeds – 18/YH/0112) and all patients will provide informed consent prior to participation, which will be obtained by study personnel with appropriate GCP training.

On completion, study results will be published in peer-reviewed journals and presented at international scientific meetings. In addition, with our PPI group, we will disseminate findings to the public, which will include lay summaries to participants and vascular charities such as the Circulation Foundation (Registered Charity Number: 1102769). The expected impact for this study is the development of a new time-efficient exercise programme for patients with IC, which is more acceptable, thus improving uptake and adherence. Should this study support the feasibility of HIIT for patients with IC, we aim to undertake a multicentre, pilot randomised controlled trial comparing HIIT to standard SEPs, which can inform a definitive trial, which has potential to impact on international guidelines.

## Supplementary Material

Reviewer comments
