# Supplementary material for: High INtensity Interval Training In pATiEnts with intermittent claudication (INITIATE): protocol for a multicentre, proof-of-concept, prospective interventional study
Source: BMJ Open. 2020 Jul 6;10(7):e038825. doi: 10.1136/bmjopen-2020-038825 (PMC7342853; doi:10.1136/bmjopen-2020-038825)
Supplement: Reviewer comments [file bmjopen-2020-038825.reviewer_comments.pdf]

PEER REVIEW HISTORY

BMJ Open publishes all reviews undertaken for accepted manuscripts. Reviewers are asked to complete a checklist review form (**http://bmjopen.bmj.com/site/about/resources/checklist.pdf**)and are provided with free text boxes to elaborate on their assessment. These free text comments are reproduced below.

ARTICLE DETAILS

**TITLE (PROVISIONAL)**	high INtensity Interval Training In pATiEnts with intermittent claudication (INITIATE): protocol for a multi-centre, proof-of-concept, prospective interventional study
**AUTHORS**	Pymer, Sean; Harwood, Amy; Ibeggazene, Said; McGregor, Gordon; Huang, Chao; Twiddy, Maureen; Nicholls, Adam; Ingle, Lee; Carroll, Sean; Long, Judith; Rooms, Marjorie; Chetter, IC

This article was not externally reviewed at BMJ Open. Protocols that have been independently assessed prior to submission to BMJ Open are usually fast-tracked to publication on the grounds that further substantial changes will not be possible. This independent assessment will usually be external, independent review for both a substantial grant award from a non-commercial or government funder and ethics approval.
